# A versatile pressure-cell design for studying ultrafast molecular-dynamics in supercritical fluids using coherent multi-pulse x-ray scattering

**DOI:** 10.1063/5.0158497

**Published:** 2024-01-03

**Authors:** Priyanka Muhunthan, Haoyuan Li, Guillaume Vignat, Edna R. Toro, Khaled Younes, Yanwen Sun, Dimosthenis Sokaras, Thomas Weiss, Ivan Rajkovic, Taito Osaka, Ichiro Inoue, Sanghoon Song, Takahiro Sato, Diling Zhu, John L. Fulton, Matthias Ihme

**Affiliations:** 1 Department of Mechanical Engineering, Stanford University, Stanford, California 94305, USA; 2 SLAC National Accelerator Laboratory, Menlo Park, California 94025, USA; 3 RIKEN SPring-8 Center, 1-1-1 Kouto, Sayo-cho, Sayo-gun, Hyogo 679-5148, Japan; 4 Chemical and Materials Sciences Division, Pacific Northwest National Laboratory, Richland, Washington 99354, USA

## Abstract

Supercritical fluids (SCFs) can be found in a variety of environmental and industrial processes. They exhibit an anomalous thermodynamic behavior, which originates from their fluctuating heterogeneous micro-structure. Characterizing the dynamics of these fluids at high temperature and high pressure with nanometer spatial and picosecond temporal resolution has been very challenging. The advent of hard x-ray free electron lasers has enabled the development of novel multi-pulse ultrafast x-ray scattering techniques, such as x-ray photon correlation spectroscopy (XPCS) and x-ray pump x-ray probe (XPXP). These techniques offer new opportunities for resolving the ultrafast microscopic behavior in SCFs at unprecedented spatiotemporal resolution, unraveling the dynamics of their micro-structure. However, harnessing these capabilities requires a bespoke high-pressure and high-temperature sample system that is optimized to maximize signal intensity and address instrument-specific challenges, such as drift in beamline components, x-ray scattering background, and multi-x-ray-beam overlap. We present a pressure cell compatible with a wide range of SCFs with built-in optical access for XPCS and XPXP and discuss critical aspects of the pressure cell design, with a particular focus on the design optimization for XPCS.

## I. INTRODUCTION

Supercritical fluids (SCFs) exhibit anomalous behaviors characterized by strong variations in thermodynamic properties, such as density, compressibility, and heat capacity, around the Widom line,^1^ representing an extension of the liquid–gas phase boundary into the supercritical regime. This thermodynamic variability makes SCFs useful for a wide range of applications in biology,^2^ food processing,^3^ material synthesis,^4^ and energy production.^5^ SCFs also occur naturally in geophysical environments, such as deep oceans,^6^ geothermal reservoirs,^7^ carbon dioxide (CO_2_) sequestration sites,^8^ and even on some extraterrestrial bodies.^9^ In Table I, we list the critical pressure, *P*_*c*_, and the critical temperature, *T*_*c*_, of some of the most common SCFs, along with their typical applications.

**TABLE I.** Critical pressure and temperature of pure fluids of practical interest. Data from NIST.^10^

Fluid	*P*_*c*_ (MPa)	*T*_*c*_ (K)	Applications
CO_2_	7.38	304	CO_2_ sequestration,
			power cycles, solvent
H_2_O	22.1	647	Subsurface reservoirs,
			chemical processing
CH_4_	4.61	191	Propulsion,
			subsurface reservoirs, solvent
Xe	5.84	290	Solvent, spacecraft propulsion
O_2_	5.04	155	Rocket propulsion
SO_2_	7.78	430	Industrial processes
C_12_H_26_	1.81	658	Rocket propulsion, jet engines
NH_3_	11.3	405	Fertilizer, refrigeration, transportation
CHF_3_	4.82	299	Organic synthesis,
			refrigeration, plasma etching
SF_6_	3.76	318	Gaseous dielectric medium
N_2_O	7.23	310	Rocket propulsion,
			internal combustion engine

Variations in thermodynamic properties of SCFs arise from their unique microscopic structure and dynamics, which is governed by inter-molecular interactions and a universal liquid–gas critical point behavior.^11^ When moving away from the critical point, the thermodynamic anomalies persist over an extended region within the supercritical regime around the Widom line, which separates the gas-like and liquid-like phase states.^12,13^ These thermodynamic anomalies are caused by self-similar cluster structures at the nano-scale.^14^ The ability to study the cluster behavior and the interaction of these clusters with solvated species is critical for advancing our fundamental understanding of SCFs and pivotal to the future development of SCF applications. This requires extensive measurements of the nano-scale structure and dynamics of supercritical fluids.

Hard x-ray and neutron scattering techniques, characterized by wavelengths shorter than 10 Å, are particularly suitable to probe these structural heterogeneities and have therefore received considerable attention over the past few decades.^15–18^ In particular, recent developments in x-ray free electron laser (FEL) light sources, capable of generating very bright femtosecond (fs) pulses, and in hard x-ray split-and-delay optics (SDO)^19–21^ have enabled novel ultrafast (fs-to-ps) two-pulse x-ray techniques, such as split-pulse x-ray photon correlation spectroscopy (sp-XPCS)^22–24^ and x-ray pump x-ray probe (XPXP). XPCS is the x-ray counterpart of dynamic light scattering and has the unique capability of studying the dynamic behavior of disordered matter at picosecond time scales and nanometer spatial scales, while XPXP measurements enable the examination of non-thermal and non-equilibrium states by perturbing the sample with a highly focused fs-x-ray pulse and measuring the subsequent microscopic structural evolution with a delayed x-ray probe pulse with femtosecond temporal resolution in the delay line. While these new coherent x-ray techniques enable measurements at unprecedented spatiotemporal resolution to examine the molecular behavior of SCFs, they introduce unique challenges that are associated with beam alignment, spatial coherence, and scattering intensity. Therefore, new SCF sample systems that are specifically tailored for these novel ultrafast multi-pulse methods are needed.

Different high pressure cells have been proposed for high-energy scattering studies of SCFs, such as diamond anvil cells^25–27^ and pressure cells.^28–43^ While diamond anvil cells are the preferred choice for achieving extreme pressures, they come with the restriction of small sample size, large pressure and temperature gradients, and limited control over pressure and temperature.^26,27^ In contrast, pressure cells provide direct control over operating conditions, excellent repeatability, and operation with different fluids and mixtures, albeit at reduced pressures compared to diamond anvil cells. Optical access for x-ray, visible, or neutron beams is provided by specialized windows.^37^ Table II provides a summary of published pressure-cell designs aimed at the study of SCFs along with their typical applications.

**TABLE II.** Pressure cell used for the study of SCFs’ nano-scale properties and structure. Expanded from Kawai *et al.*^37^ Be: beryllium; Be-PEEK: beryllium-reinforced polyether ether ketone; BS: window fixed using a Bridgman seal; BW: brazed window; c-BN: cubic boron nitride; DLS: dynamic light scattering; EH: electrically heated; GPS: window fixed using a Poulter seal, initially affixed with adhesive glue; IXS: inelastic x-ray scattering; PC: pressure cell; P-jump: pressure jump; PS: window fixed using a Poulter seal; SR: stirred reactor; SAXS: small-angle x-ray scattering; SPS: window fixed using a spring loaded Poulter seal; TRIR: time-resolved infrared absorption spectroscopy; WAXS: wide-angle x-ray scattering; WH: water heated; and XAS: x-ray absorption spectroscopy.

References	Method	Cell type	Window material	Window thickness	Aperture	Fluid	Operating conditions
Nishikawa and Takematsu^28^	SAXS	WH PC with BW	Be	2.0 mm	8.5 mm	CO_2_	10 MPa, 333 K
Pfund *et al.*^29^	SAXS	EH PC with BW	Diamond	500 *µ*m	3.0 mm	Solutions in sCO_2_, Xe	50 MPa, 319 K
Nishikawa and Morita^30^	SAXS	WH PC with PS	Diamond	400 *µ*m	4.0 mm	CHF_3_	15 MPa, 333 K
Hoffmann *et al.*^31^	TRIR	EH PC with Pt washer seals	Laser drilled diamond	1.0 mm	5.0 mm	Solutions in sH_2_O/sCO_2_	38 MPa
							673 K
Koga *et al.*^32^	SAXS, DLS	EH PC with SPS	Diamond	500 *µ*m	2.0 mm	Solutions in sCO_2_	70 MPa
Fulton *et al.*^33^	XAS	EH PC with GPS	Diamond	25 *µ*m	300 *µ*m	Solutions in sH_2_O/sCO_2_	100 MPa
							773 K
Testemale *et al.*^34^	XAS, SAXS	Cold wall	Be	1.5 mm	⋯	Aqueous solutions	200 MPa
	IXS	He furnace	Sapphire	600 *µ*m	⋯		873 K
Grunwaldt *et al.*^35^	XAS	EH PC with stirrer	Be-PEEK	500 *µ*m	⋯	Catalysis in sCO_2_	25 MPa, 493 K
Ando *et al.*^36^	SAXS	PC with PS	Diamond	500 *µ*m	1.2 mm	Protein in H_2_O	400 MPa, 300 K
Kawai *et al.*^37^	XAS	EH PC with BW	c-BN	800 *µ*m	3.0 mm	Catalysis in H_2_	10 MPa, 900 K
Brooks *et al.*^38^	SAXS	WH P-jump PC with GPS, BS	Diamond	1.0 mm	2.5 mm	Soft condensed matter	500 MPa
	WAXS						393 K
Hermida-Merino *et al.*^39^	S/WAXS, XAS	EH SR PC with BW	Diamond	400 *µ*m	4 mm	Solutions in sCO_2_	21 MPa, 393 K
Skinner *et al.*^40^	WAXS	EH PC with PS	Diamond	250 *µ*m	⋯	H_2_O	360 MPa, 300 K
Rai *et al.*^41^	SAXS	WH PC with BS	Diamond	500 *µ*m	1.5 mm	Solutions in H_2_O	400 MPa, 353 K
Miller *et al.*^42^	SAXS	PC with BS	Diamond	500 *µ*m	1.5 mm	Protein in H_2_O	400 MPa, 350 K
Present	SAXS, WAXS,	EH PC with GPS	Diamond	100 *µ*m	1.0 mm	H_2_O, CO_2_, Xe	30 MPa
	sp-XPCS, XPXP						
							675 K

By leveraging prior work on pressure cells for x-ray-based measurements, here we present a pressure cell that is specifically designed for multi-pulse FEL experiments. This pressure cell has been demonstrated at pressures up to 30 MPa and temperatures up to 675 K, thereby enabling the examination of supercritical water and all other fluids listed in Table I. By considering particularly stringent requirements on the intensity, speckle contrast, and signal-to-noise ratio (SNR), the pressure cell is primarily optimized for sp-XPCS experiments. The flexible design also enables experiments with more conventional x-ray techniques, such as XPXP and small/wide angle x-ray scattering (S/WAXS). So far, this cell has been successfully used for sp-XPCS experiments at the Linac Coherent Light Source (LCLS, SLAC National Accelerator Laboratory, Menlo Park, CA, USA), for XPXP experiments at the SPring-8 Angstrom Compact-Free Electron Laser (SACLA, SPRING-8, Koto, Sayo, Hyogo prefecture, Japan), and for S/WAXS experiments at the Stanford Synchrotron Radiation Lightsource (SSRL, SLAC National Accelerator Laboratory, Menlo Park, CA, USA).

The remainder of this paper has the following structure. In Sec. II, we summarize essential sp-XPCS theory to guide the design of the pressure cell. In Sec. III, we present the detailed design, dimensions, methods, and suppliers used to manufacture the pressure cell. With relevance to ensuring repeatability for high temperature conditions, this section also discusses the assembly procedure that we found to yield optimal stability and performance. The thermal and mechanical performance of the cell is then characterized in Sec. IV, and conclusions are presented in Sec. V.

## II. BACKGROUND ON SP-XPCS THEORY

**FIG. 1.**
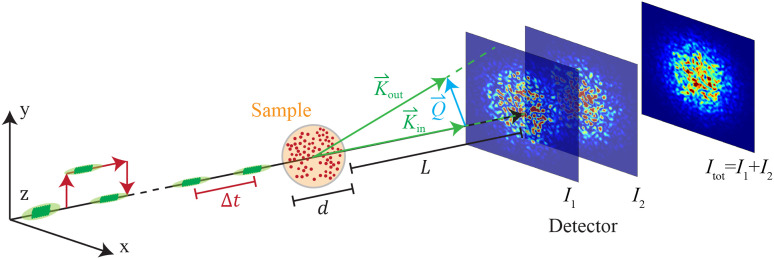
Operating principle of sp-XPCS. *d* and *L* designate the sample thickness and sample-to-detector distance, respectively; ${\vec{K}}_{in}$ is the central wave-vector of the incident x-ray pulse pair; ${\vec{K}}_{\mathrm{out}}$ is defined for each detector pixel and is the central wave-vector of the scattered x-ray pulse, pointing from the sample to each individual pixel in the detector; $\vec{Q}={\vec{K}}_{\mathrm{out}}-{\vec{K}}_{in}$ and $Q=|\vec{Q}|$ are defined for each pixel on the detector; and *I*_1_ and *I*_2_ represent the speckle pattern intensity of the individual pulses on the area detector. At fs-to-ps time separation between pulses (Δ*t*), the area detector cannot separate the two diffraction patterns arising from each individual pulse and only records the total diffraction pattern *I*_tot_ = *I*_1_ + *I*_2_.

XPCS is the counterpart of dynamic light scattering at hard x-ray wavelengths and measures the sample dynamics through the intermediate scattering function (ISF),^44^ *f*(*Q*, Δ*t*). Here, *Q* is the angular wave number of the corresponding length scale under investigation and Δ*t* is the sample evolution time, and its definition in the scattering geometry in shown is Fig. 1.

For sample dynamics on fs-to-ps time scale and nm length scale, the ISF can be measured using the sp-XPCS technique, the schematic of which is shown in Fig. 1. In sp-XPCS, a fs x-ray FEL pulse is split into a pulse pair with a SDO,^21^ and both x-ray pulses are sent to the same location on the sample along the same direction with a controlled relative delay time Δ*t* between the two pulses. This pulse pair produces a complex interference pattern on the area detector, illustrated in Fig. 1, and is referred to as a speckle pattern. The sharpness of the speckle pattern, quantified by its contrast *β*, is influenced by the atomic motion in the sample during the interval Δ*t* between the pulses in the pair. Therefore, by determining the speckle contrast *β* for different Δ*t*, one can measure the ISF and extract information about the sample dynamics.

The definition of the speckle contrast and its relation to the ISF in sp-XPCS is^45^

$$\begin{array}{ll} \beta \left(Q,\Delta t\right) & \equiv \frac{〈I_{\text{tot}}^{2}〉}{{\left\langle I_{\text{tot}}\right\rangle }^{2}}-1=\beta_{1}r^{2}+\beta_{2}{\left(1-r\right)}^{2}\\ & \;+2\mu r\left(1-r\right)\min\left(\beta_{1},\beta_{2}\right)f\left(Q,\Delta t\right), \end{array}$$ (1)

where $\beta \left(Q,\Delta t\right)$ is the contrast of the speckle pattern from the pulse pair at a specific angular wave number *Q* and a specific relative delay time Δ*t* of the pulse pair. *I*_tot_ is the scattering intensity from the pulse pair at a detector pixel with an angular wave number of *Q* and *I*_tot_ = *I*_1_ + *I*_2_, where *I*_1_ and *I*_2_ are scattering intensity contributions of each single pulse in the pulse pair. The angular bracket ⟨·⟩ indicates the average value of the quantity over a series of x-ray pulses. *r* = ⟨*I*_1_⟩/⟨*I*_tot_⟩ is the average intensity ratio between the two pulses in the pulse pair. *β*_1_ and *β*_2_ are the speckle contrasts if only one pulse in the pulse pair is diffracted by the sample. $\mu \in \left[0,1\right]$ measures the effective overlap between the two pulses on the sample and is sensitive to both spatial and angular overlap of the two pulses.

The effective overlap *μ* has significant influence on the sp-XPCS measurement sensitivity and is affected by both the SDO design and its alignment. With the latest grating-based SDO design,^21^ almost identical hard x-ray pulse pairs, *β*_1_ ≈ *β*_2_, can be generated with *μ* ≥ 0.9 over an extended range of delay times $\Delta t\in \left[0,10\right]$ ps. However, due to x-ray source and optics drifts, such a high *μ* value cannot be maintained over an entire measurement, which usually lasts more than 6 h. A realignment and geometric optimization of the SDO is therefore needed approximately every 30 min to maintain this high *μ* value.^21^ Therefore, in Sec. III D, we present our design of a high resolution beam profile monitoring system tailored for the pressure cell to optimize x-ray pulse overlap *μ*.

In practice, the x-ray photon shot noise is another major source of uncertainty for the measurement of the speckle contrasts *β*, *β*_1_, and *β*_2_. Usually millions of speckle patterns need to be accumulated to reach a signal-to-noise ratio (SNR) of the contrast measurement *β* that is sufficiently high to discern the variation of *f*(*Q*, Δ*t*) as a function of Δ*t*. The SNR of *β* has a complex dependence on the sample thickness, *d*, and the distance between sample and detector, *L*, through the contrast *β* itself and the mean scattering intensity *I*. Choosing *d* and *L* for an optimal SNR of *β* can greatly improve the measurement efficiency in sp-XPCS.

At a low photon count rate, *I* ≤ 10^−2^ photon/pixel/pattern, which is common for sp-XPCS on SCFs, the shot-noise-induced SNR can be estimated as^46^

$$\text{SNR}\equiv \frac{\beta }{\sigma_{\beta }}\approx I\beta {\left(\frac{N_{\text{pixel}}N_{\text{pattern}}}{2\left(1+\beta \right)}\right)}^{\frac{1}{2}},$$ (2)

where *σ*_*β*_ is the standard deviation of the estimation of the speckle contrast *β*, *I* is the average photon count rate, *N*_pixel_ is the number of pixels within the region of interest on the area detector, and *N*_pattern_ is the number of patterns collected. Equation (2) can be used to estimate the shot-noise-induced SNR for *β*, *β*_1_, and *β*_2_. In each case, *I* is the corresponding mean scattering intensity ⟨*I*_tot_⟩, ⟨*I*_1_⟩, and ⟨*I*_2_⟩.

The scattered x-ray intensity at the detector per pixel and per pulse is given as

$$I=I_{0}\;d\;\exp\left[-\frac{d}{d_{\mathrm{att}}}\right]{\left(\frac{d\sigma }{d\Omega }\right)}_{\text{Th}}d\Omega ,$$ (3)

where *I*_0_ is the incident x-ray photon flux, (*dσ*/*d*Ω)_*Th*_ is the differential cross section for Thompson scattering, and *d*Ω is the solid angle of the pixel with respect to the sample. *d*_att_ is the x-ray attenuation length, which depends on the sample composition and density. (*dσ*/*d*Ω)_Th_ is determined from S/WAXS measurement, described in Appendix B, and is dependent on the sample composition, pressure, and temperature, as well as the angular wave number *Q*.

For a specific x-ray light source, by reducing *d* or increasing *L*, one increases the contrast *β*.^45^ This, however, reduces the scattering intensity *I*, as shown in Eq. (3). According to Eq. (2), the net influence on the SNR of *β* depends on their product. One therefore needs to thoroughly examine the feasible experimental parameter space for each specific sample to find the optimal values for the geometric parameters *d* and *L*.

**FIG. 2.**
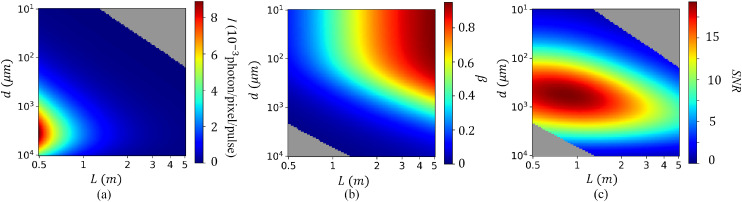
Determination of optimal experimental parameters for sp-XPCS experiments at *Q* = 0.1 Å^−1^ with supercritical water at 25 MPa and 653.15 K. The geometrical parameters of interest are the sample thickness *d* and the sample-to-detector distance *L*. (a) Scattering intensity *I* at the detector, (b) single pulse speckle contrast *β*_1_, and (c) shot-noise-induced SNR of the contrast measurement with *N*_pattern_ = 4.3 × 10^5^ diffraction patterns, corresponding to 1 h of data acquisition at a 120 Hz FEL repetition rate at LCLS. The gray region represents conditions of *I* and *β* in which current state-of-the-art analysis tools cannot reliably extract physical information. The boundaries of these inaccessible regions are *I* < 10^−5^ photon/pixel/pattern in (a) and *β* < 0.01 in (b). Experimental conditions: 50 *µ*m detector pixel size, 9.5 keV photon energy, 3 *µ*m beam diameter, 3 × 10^8^ photon per pulse, 0.4 eV FWHM energy bandwidth, and full transverse coherence.

In the following, we estimate the optimal geometric parameters *d* and *L* to perform sp-XPCS measurements on supercritical water at 25 MPa and 653 K, considering specifically the angular wave number *Q* = 0.1 Å^−1^. The FEL operating conditions are representative of the XPP instrument at LCLS, with an x-ray photon energy of 9.5 keV, an energy full-width-half-maximum (FWHM) bandwidth of Δ*E* = 0.4 eV, an x-ray beam size of 3 *μ*m, an incident x-ray photon flux of *I*_0_ = 3 × 10^8^ photons per pulse on the sample, and an x-ray repetition rate of 120 Hz. The detector pixel size is assumed to be 50 *µ*m, corresponding to an Epix100 detector.^47^ The results of the analysis are shown in Fig. 2. Figure 2(a) shows the scattering intensity at the detector per pixel as a function of sample thickness *d* and detector distance *L* using Eq. (3). Figure 2(b) shows the single pulse speckle contrast as a function of *d* and *L*. We have assumed a Gaussian spatiotemporal beam profile of the x-ray pulse with x-ray beam parameters specified above to calculate the speckle contrast *β* following the derivations given in Refs. 45 and 48.

Limited by the background noise level of modern hard x-ray area detectors and numerical instabilities in current state-of-the-art analysis algorithms, if the x-ray scattering *I* is too weak (*I* < 10^−5^ photon/pixel/pattern) or if the single pulse speckle contrast *β* is too low (*β* < 0.01), the speckle contrast analysis will be heavily dominated by photon shot noise and systematic error from the analysis algorithm and therefore cannot be used to measure the sample dynamics. In Fig. 2, we have marked these inaccessible regions of the geometric parameter space in gray.

In Fig. 2(c), we plot the SNR as a function of *d* and *L*, assuming 1 h of continuous data acquisition with a 120 Hz FEL repetition rate following Eq. (2). The pixel number *N*_pixel_ as a function of sample-detector distance *L* is calculated according to the model described in Appendix C. As shown in Fig. 2(c), within the feasible region (non-gray region), the maximum SNR for sp-XPCS measurements is achieved for a sample thickness of *d* ≈ 0.8 mm and detector distance *L* ≈ 1 m. For these geometric parameters, 1 h of data acquisition yields an SNR = 18.8. If we assume that the SDO is optimized such that *r* ≈ 0.5 and *μ* ≈ 1, then SNR = 18.8 guarantees that a 5% change in *f*(*Q*, Δ*t*) will be statistically significant. Following the same procedure, systematic SNR analysis can be conducted for different pressures, temperatures, angular wave numbers, and evolution time to plan experiments and optimize the sensor and sample geometries ahead of a beamtime.

We emphasize here that for different sample compositions, sample thermodynamic conditions, x-ray beam characteristics, and detector specifications, the optimal sample thickness *d* can vary significantly in a range spanning from tens of micrometers to several millimeters, since the thermodynamic condition and angular wave-vector *Q* of the sample determine its differential cross section in Eq. (3) and the elastic scattering intensity *I* on the detector. Therefore, in the pressure cell design presented in this paper, we integrate a flexible procedure to adjust the sample thickness by changing a single component, allowing us to vary the sample thickness in the range *d* ∈ [200 *μ*m, 3 mm]. Due to deformation under high temperature and pressure conditions and unavoidable assembly uncertainties, the sample thickness during the experiment can differ from its design value. We therefore measure the sample thickness before conducting any experiment. The procedure to perform these measurements is presented in Sec. IV B and Appendix F.

## III. PRESSURE CELL

The pressure cell presented in this study is adapted from previous designs introduced in Table II and combines several features to improve its flexibility and suitability for multi-pulse x-ray experiments. The demonstrated working temperature and pressure of this pressure cell are 675 K and 30 MPa.^49^ These conditions are chosen to enable experiments with a variety of fluids of practical relevance, including water and dodecane, which both have a high critical temperature (Table I). To facilitate optical experiments at both x-ray and visible wavelengths, we employ large optical windows and large accessible scattering angles so that this pressure cell can be utilized at a variety of x-ray beamlines. The key features of this pressure cell are (i) variable sample thickness, (ii) metal-to-metal seal for high temperature operation, (iii) thin diamond windows to reduce background scattering, and (iv) integrated diagnostics to enable *in situ* x-ray beam monitoring. The computer aided design (CAD) files for the pressure cell are provided in STEP format as the supplementary material.

### A. Pressure-cell design and manufacturing

**FIG. 3.**
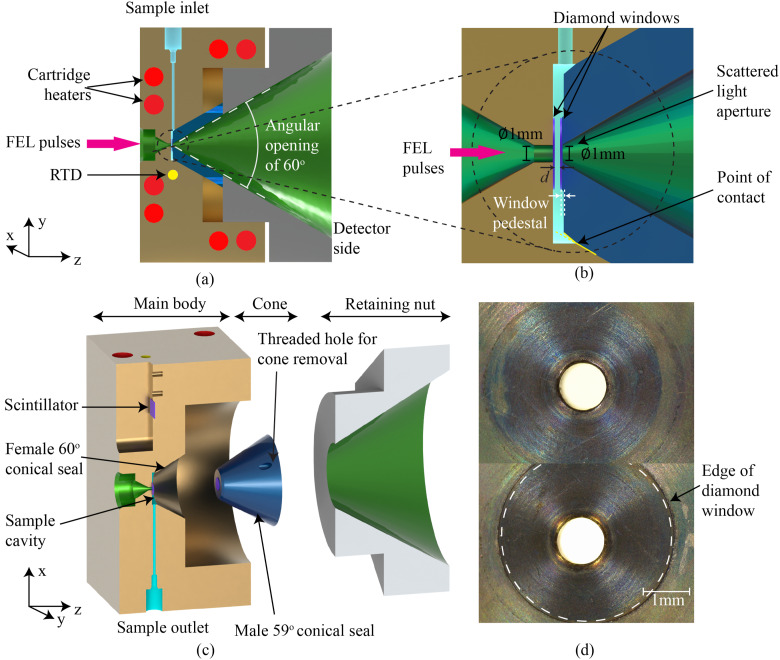
3D model of the versatile pressure cell. (a) Cut view showing the fully assembled pressure cell and the integration of the heating system. The cutting plane used in (a) is orthogonal to that used in (c); (b) zoomed-in view of the sample cavity, optical access apertures, and diamond windows; (c) exploded cut view showing the four main components of the cell assembly: main body, cone, retaining nut, and scintillator; and (d) magnified view of the window support surface (top) and the assembled diamond window (bottom) acquired under a Leica S9i microscope (Deerfield, IL, USA). (a)–(c) The FEL pulses are traveling from left to right.

Figure 3 shows the pressure cell and its main features. This pressure cell assembly consists of four main components [Figs. 3(a) and 3(c)]: the main body [golden brown in Fig. 3(c)] supporting the upstream x-ray window, a cone [dark blue in Figs. 3(a) and 3(c)] supporting the detector-side x-ray window and sealing the sample cavity when pressed against the main body, a retaining nut [gray in Figs. 3(a) and 3(c)], and a scintillator assembly [dark purple in Fig. 3(c)]. The sample cavity is shown in Fig. 3(b), and the optical paths of the FEL pulses and the scattered light are shown in green in Figs. 3(a)–3(c). All components are machined in-house at the SLAC National Accelerator Laboratory from grade 5 Ti-6Al-4V titanium alloy, selected for its mechanical properties at high-temperature and its proven chemical inertness with supercritical water and supercritical aqueous solutions.^50,51^

The main body is a square cuboid with external dimensions of 40.5 × 76.2 × 76.2 mm^3^. Located at the center of the main body, facing the light source, is the laser/x-ray aperture, a 1 mm-diameter hole leading to the sample cavity. In order to allow visual inspection and monitoring of the window, this aperture is placed at the end of a viewing port. The viewing port consists in a 9 mm-deep unpolished 60° conical bore. An off-axis high resolution imaging system, presented in Sec. III D, is attached to the cell to visually inspect the windows and the sample during experiments. The sample cavity is an open-end 10 mm-diameter 1.58 mm-deep cylinder, shown in cyan in Figs. 3(a) and 3(b), and has separate inlet and outlet sample lines, also shown in cyan in Figs. 3(a) and 3(c). The inlet and outlet lines are drilled perpendicular to each other, which allows for bleeding off any air present in the sample cavity during initial filling of the cell. It also enables the operation of this pressure cell as either a hydrostatic cell or a flow cell. The sample feed lines are 0.8 mm in diameter and are terminated by 1/16 in. high pressure taper seal grade 5 titanium tube fittings from High Pressure Equipment (Erie, PA, USA). Extending from the open end of the cylindrical sample cavity is a 60° 8.66 mm-deep conical section. At the downstream end of the main body is a 50 mm-diameter 20.2 mm-deep M50 × 1.5 fine-pitched threaded hole that the retaining nut is threaded into during operation.

The main body of the pressure cell assembly also serves as a heat bath for the SCF sample. Heat is supplied by eight cartridge heaters located in the bores shown in red in Fig. 3(a). The temperature of the main body is monitored using a resistance temperature detector (RTD), shown in yellow in Fig. 3(a), and whose sensing element is placed near the sample cavity.

To assess the alignment of the x-ray pulses on the sample during sp-XPCS and XPXP experiments, a x-ray scintillator screen, made of YAG crystals, is installed at the edge of the main body [Fig. 3(c)]. The upstream surface of the YAG screen is coplanar with the mid-plane of the sample cavity and, therefore, only requires a short transverse translation during experiments to measure the pulse overlap on the sample.

The cone [dark blue in Fig. 3(c)] is inserted into the main body on the detector side and pressed in place using the retaining nut. It seals the sample cavity by forming a metal-to-metal swaged seal with the main body. That is achieved by having a slight mismatch between the angle of the female conical bore on the main body (60°) and the male conical surface of the cone (59°), as illustrated in Fig. 3(c). The metal-to-metal contact between the two parts then occurs along a single circular line, forming a seal. Both conical surfaces are manufactured using single-point diamond turning^52^ to obtain an accurate geometry and fine surface finish. When pressed together by tightening the retaining nut, the two conical surfaces deform slightly at the contact line and create a very reliable swaged seal capable of operating at high pressure and high temperature.^53^ At the contact line between the male and female parts, there is a small step characterized by a change in the angle of the male cone, emphasized in Fig. 3(b) with a yellow line. This small step allows us to more precisely set the location at which the two conical parts mate and ensures that the positioning of the cone is repeatable. Multiple cones were manufactured with different step heights, providing an affordable approach to adjust the sample thickness *d* to specific experimental conditions and optimize the SNR for XPCS and XPXP measurements. The laser/x-ray light passes through a 1 mm diameter aperture at the center of the cone. An inner 60° conical bore [shown in green in Fig. 3(a)] is also machined in the cone to transmit photons scattered at large angles toward the detector.

The retaining nut is hexagonal, with an overall length of 35 and a 76.3 mm width. It has a 15 mm-long M50 × 1.5 fine pitch thread to screw into the main body of the cell. The thread was selected to apply sufficient torque to preload the swaged conical seal and to have a high mechanical strength.^38^ The threading on the retaining nut is the major potential failure point of this design. We performed a pressure safety analysis to guarantee a safety factor of 11.3 for the retaining nut threading with a maximum working pressure of 30 MPa. The analysis is summarized in Appendix A. The retaining nut also has an internal conical bore with a 60° included angle to transmit scattered x-ray photons over a large angular range. A small amount of Silver Goop high temperature lubricant (Swagelok, Solon, OH, USA) can be applied to the threads for lubrication.

To achieve fast, safe, and reproducible installation and removal of the pressure cell in the case of unexpected incidents during experiments, a customized installation interface between the pressure cell and beamline infrastructure is used. Details of this interface are presented in Appendix D.

### B. X-ray windows and their installation and removal

X-ray optical access is provided by two single-crystal, type IIa, diamond windows, 100 *µ*m thick and 4 mm in diameter (Applied Diamond, Wilmington, DE, USA). The thickness needs to be minimized to reduce the scattering background of the windows, and the optimal thickness was determined using the following equation:^38^

$$p_{\max}=\frac{4}{3\sqrt{1-\nu +\nu^{2}}}{\left(\frac{t}{a}\right)}^{2}\sigma_{y},$$ (4)

where *p*_max_ is the maximum sample pressure before failure, *t* is the window thickness, *a* is the unsupported aperture radius, and *σ*_*y*_ and *ν* are the yield strength and Poisson ratio of type IIa diamond. The material properties of the diamond window are provided in Appendix G. The 100 *µ*m window thickness provides a safety factor of 1.9 at the maximum design pressure of 30 MPa. During an over-pressurization test, we observed a diamond window failure at ∼690 bars, larger than the theoretical value estimated using Eq. (4). At the typical hard x-ray energy of 10 keV used at LCLS and SACLA for sp-XPCS experiments, each window transmits 92% of the incident pulses.

**FIG. 4.**
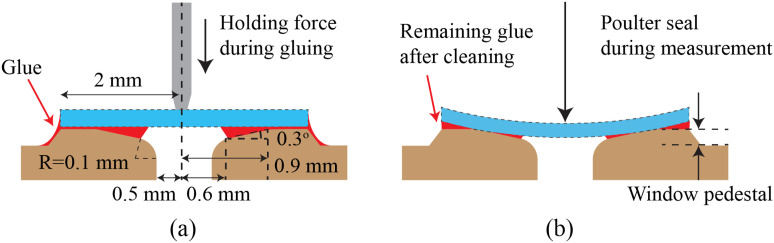
Procedure for installing diamond window in the pressure cell: (a) apply moderate pressure on the diamond window and apply adhesive along the outer rim with a thin brush and (b) during operation, fluid pressure on the sample deforms the diamond window and forms a Poulter seal. Not to scale.

Given the high operating temperature and pressure, the thin diamond windows are attached to the cell with a Poulter seal.^54^ With this design, the diamond window rests on a highly polished metal seat. Following the design recommendations by Sherman and Stadtmuller,^53^ the seats of the Poulter seal are placed on small pedestals [Fig. 3(b)] that are not perfectly flat but form a shallow cone with a 0.3° angle between 0.6 ≤ *r* ≤ 0.9 mm from the center of the aperture, as illustrated in Fig. 4(a). The metal surfaces supporting the diamond windows are machined with the single-point diamond turning technique, yielding an optically reflective surface finish with a precise geometry and a circular lay without the need for additional polishing. During operation, the pressure within the sample cavity deforms the seat and window, forming a tight seal capable of operating at very high pressures, as shown in Fig. 4(b).

A Poulter seal, although highly effective at high pressures, does not seal at ambient or low-pressure conditions (*P* ≲ 2 MPa). A typical solution to this issue is to affix the window onto its supporting surface. However, at and above the critical temperature of water, most adhesives melt and ultimately contaminate the sample and the optical path. In what follows, we briefly describe an affixing procedure that we found effective at minimizing issues associated with adhesive contamination, particularly when conducting experiments at high temperatures.

We selected a high vacuum sealant, Vacseal (original formulation, Space Environment Labs, Boulder, CO, USA), for its high operating temperature and low viscosity. We start by thoroughly cleaning the surfaces of the Poulter seal and diamond with acetone. The diamond window is then placed and centered onto its seat and clamped using either a soft wood rod or a vacuum tweezer. A thin brush is then used to deposit a small amount of adhesive on the metallic seat of the Poulter seal, along the outer rim of the diamond window. Capillary forces will allow the low-viscosity adhesive to wick into the gap between the diamond window and the metal seat of the seal. We found that the direct application of the adhesive between the diamond window and the metal surface would result in excess adhesive being present, which would inevitably leak into the sample or the beam path during high-temperature experiments. The cone, pressure cell body, and diamond windows are then oven-cured at 300 °C for 30 min. After curing and cooling the pressure cell, excess adhesive is cleaned by placing the cone and pressure cell body in an ethanol bath, within an ultrasonic cleaner (Branson Ultrasonics Corp., Danbury, CT, USA) for 15 min. The ultrasonic cleaning can significantly weaken the excess adhesive exposed to the ethanol but will not break the bond between the diamond and its metal support. The excess adhesive can then be removed with a cotton tip applicator.

To break the adhesive bonds between the diamond window and the Poulter eat after the completion of an experiment, the parts are placed in an acetone bath within an ultrasonic cleaner for 15 min.

### C. Pressure and temperature control

**FIG. 5.**
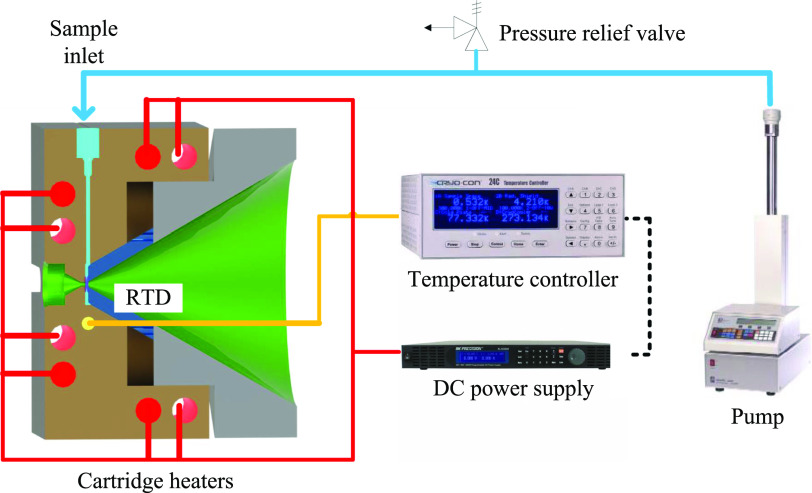
Piping and instrumentation diagram showing the arrangement of the fluid delivery system and the heating stage.

Figure 5 shows a diagram of the pressure and temperature control system used for the operation of this cell. A syringe pump (Teledyne ISCO 100DM, Lincoln, NE, USA) is used for fluid delivery and pressurization. The pressure in the cell is regulated by a proportional-integral-derivative (PID) controller driven by the pump’s internal pressure sensor. An additional redundant pressure sensor is placed near the pressure cell, achieving a measurement accuracy of the absolute pressure of the sample of ±0.12 MPa. During extended continuous operation of the pressure, pressure stability was measured to be better than ±0.02 MPa over 60 h, which is further discussed in Sec. IV A. A pressure relief valve (Swagelok, Solon, OH, USA) is added to the system for safety and compliance with pressure vessel regulations.

The temperature of the pressure cell is monitored with a 1/4 in. diameter 100Ω platinum resistance temperature detector (Omega Engineering, Norwalk, CT, USA) whose location is shown in yellow in Fig. 3(a). The temperature signal is picked up by a temperature controller (CryoCon Model 24C, Cryogenic Control Systems, Rancho Santa Fe, CA, USA), which is executing a PID control loop to stabilize the temperature. The control signal drives the voltage output of an external DC power supply (BK Precision, Yorba Linda, CA, USA), which provides electric power to the eight cartridge heaters (Briskheat, Columbus, OH, USA) on the pressure cell, each rated at 250 W.

To reduce the heat loss and to improve temperature stability, the pressure cell is thermally insulated using high-temperature ceramic paper (Fiberfrax 970, Unifrax, Tonawanda, NY, USA). In addition, the cell is separated from beamline components with four 9.5 mm-outer-diameter 12.7 mm-long grade L5 ceramic posts (McMasterCarr, Elmhurst, IL, USA) to minimize metal-to-metal heat losses.

An additional temperature probe is placed inside the sample cavity, in direct contact with the SCF, to provide a redundant and more accurate temperature measurement. A small high-accuracy 0.5 mm-diameter K-type thermocouple probe (Omega Engineering, Norwalk, CT, USA) was used for this purpose. It is inserted into the sample cavity through the tubing of the sample inlet line.

### D. X-ray alignment and sample motion

**FIG. 6.**
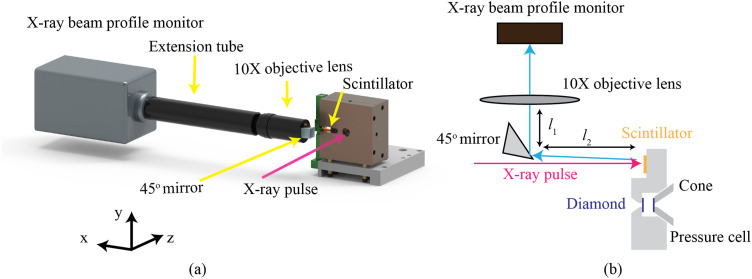
(a) Imaging system with a high-resolution camera, a right-angle mirror, and a scintillator screen; (b) schematics of the optical path of the diagnostic system. *l*_1_ and *l*_2_ measure the distance from the lens to the mirror and from the mirror to the scintillator screen, respectively. The total distance, *l*_1_ + *l*_2_ = 51 mm, corresponds to the working distance of the objective lens.

For multi-pulse x-ray measurements, such as XPXP and sp-XPCS, the spatial and angular alignment of x-ray pulses is crucial to optimize the SNR of the measurements [Eq. (1)]. For hard x-ray pulses at FELs, the focused x-ray beam size typically used for such measurements is on the order of 1–3 *µ*m. Due to inherent electron and device instability, it is common practice to check and if necessary adjust the x-ray beam overlap at 30 min time-intervals during operation.^21^ To reduce the time associated with beam monitoring and alignment, we designed a dedicated imaging system to facilitate this process, which is shown in Fig. 6. In this setup, a right angle mirror (12.5 *μ*m protected gold coated N-BK7 right angle mirror, Edmund Optics, Barrington, NJ, USA) is installed tangent to the x-ray beam path. A high magnification camera assembly (ZYLA-5.5-USB3, Andor, Belfast, UK), with a 152.5 mm extension tube and a 10× Mitutoyo (Kanagawa, Japan) telecentric microscope objective with a spatial resolution of 325 nm, is used to image the x-ray beam profile on the scintillator attached to the side of the cell, shown in yellow in Fig. 6. The scintillator screen is a 5 mm × 5 mm × 20 *µ*m YAG crystal and is mounted within a slot machined in the main body of the cell.

The bottom of the slot is parallel to and located 300 *µ*m downstream to the front surface of the sample cavity. This defines the relative position between the scintillator screen with respect to the sample cavity. Therefore, after optimizing the x-ray optics alignment with the scintillator screen, we can move the pressure cell to compensate the relative position between the scintillator and the sample cavity, ensuring optimal x-ray overlap and placing the focus spot in the middle of the sample cavity. A motorized translation is placed on the beamline to switch between the sample and scintillator. In Appendix E, we present in more detail the motion system required to achieve an efficient and precise sample alignment and a rapid sample-scintillator translation.

## IV. CHARACTERIZATION

### A. Pressure and temperature envelope

**FIG. 7.**
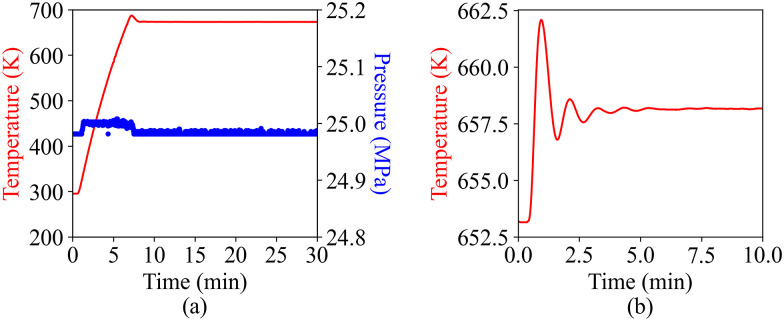
Temperature (red) and pressure (blue) for different operating conditions: (a) transient heating of the sample from room temperature (295.32 K) to 673.15 K at 25 MPa at a heating rate of 54.2 K/min and (b) changing of sample temperature from 653.15 to 658.15 K at 25 MPa.

The thermal stability and step-response characterization of the pressure cell is presented in Fig. 7. The test was conducted with a 500 *μ*m-thick supercritical water sample. Prior to the test, the PID parameters of the temperature controller were optimized using its built-in auto-tune functionality for a target temperature of 673.15 K. Figure 7(a) shows the temperature and pressure time profiles during initial heating of the water sample from room temperature (295.32 K) to the target temperature of 673.15 K with a nominal pressure of 25 MPa. A heating rate of 54.2 K/min is achieved, and after 15 min, the temperature stabilizes within 0.2 K of the target. Figure 7(b) shows the thermal response for a change of the setpoint temperature from a steady state of 653.15–658.15 K. After an overshoot to 662.08 K at *t* = 1 min, the temperature stabilizes to 658.15 K within 0.1 K of the target in less than 5 min. Over an extended measurement of 60 h at steady state, we are able to maintain a constant pressure with less than 0.017 MPa peak-to-peak fluctuations and a constant temperature with less than 0.22 K peak-to-peak fluctuations.

During operation with supercritical water, the leakage rate of the cell is less than 9 *μ*l h^−1^. During x-ray experiments, to avoid accumulated effects of sample irradiation, such as heating and ionization, we typically operate the cell as a flow cell with a low flow rate.

### B. Sample thickness

For XPXP and sp-XPCS measurements, the sample thickness has a significant impact on the SNR. We therefore characterize the sample thickness at the beginning of each experiment using x-ray attenuation. This technique requires independent measurements of the intensities of the incident (*I*_0_) and transmitted (*I*_1_) x-ray pulses at different sample densities.

**FIG. 8.**
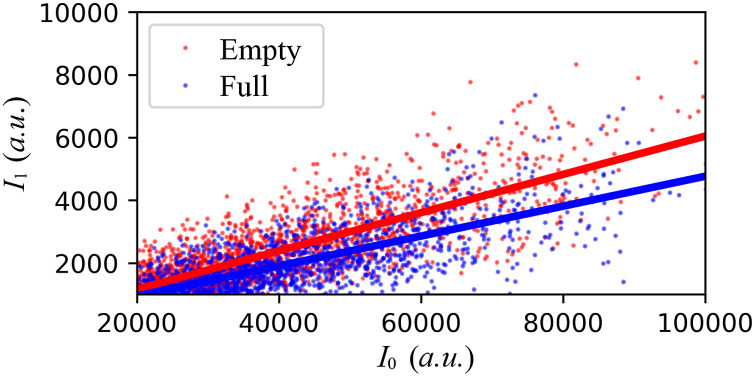
Correlation plot of the transmitted x-ray intensity *I*_1_ against the incident x-ray intensity *I*_0_ for two sample conditions: *empty* and *full*. One can derive the sample thickness following Eq. (5).

Assuming that the attenuation length of the sample is *ξ*_1_ and the sample thickness is *d*, we have $I_{1}=\eta I_{0}\;\exp\left\{-d/\xi_{1}\right\}$, with *η* being a constant representing the x-ray energy loss unrelated to sample attenuation. Upon repeating the measurement with different sample densities and therefore different attenuation lengths, *ξ*_2_, the sample thickness can be evaluated as

$$d=\frac{\xi_{1}\xi_{2}}{\xi_{1}-\xi_{2}}\left(\ln\tau_{1}-\ln\tau_{2}\right),$$ (5)

$$\Delta d=\frac{\xi_{1}\xi_{2}}{\xi_{1}-\xi_{2}}{\left[{\left(\frac{\Delta \tau_{1}}{\tau_{1}}\right)}^{2}+{\left(\frac{\Delta \tau_{2}}{\tau_{2}}\right)}^{2}\right]}^{\frac{1}{2}},$$ (6)

with *τ*_*i*_ ≡ *I*_*i*_/*I*_0_ for *i* = {1, 2}. Representative measurements are shown in Fig. 8 for a cell configuration with a nominal sample thickness of 1 mm. In Fig. 8, the slope of each line corresponds to *τ*_*i*_. Following Eq. (5), one can readily derive the actual sample thickness as 954 ± 108 *µ*m. The attenuation measurement of the sample thickness is not limited to x-ray pulses and can also be carried out using a visible wavelength laser, as further discussed in Appendix F.

### C. Signal-to-background analysis and x-ray scattering experiments

In x-ray scattering experiments that are conducted with samples contained in pressure cells, it is important to minimize the scattering from the cell’s windows and achieve a high signal-to-background ratio. For different types of x-ray scattering techniques, the background level can vary significantly due to geometric constrains. Since our pressure cell operates in the atmosphere, scattering from the air and diamond windows contribute primarily to the background scattering. In this section, we present comparisons between signal and background scattering levels for sp-XPCS and XPXP experiments. In addition, measured quantities for studying microscopic dynamics in SCFs are also presented in this section.

**FIG. 9.**
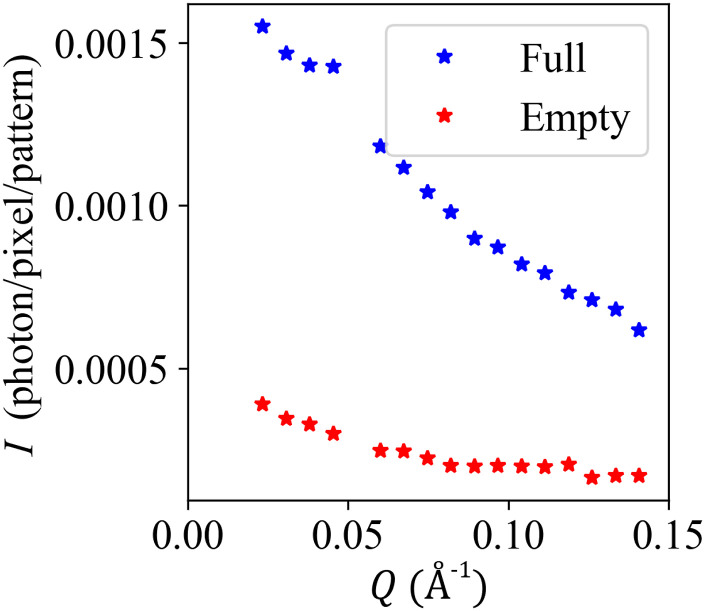
Mean scattering intensity, *I*, at different scattering angular wave numbers, *Q* (Å^−1^), from the empty pressure cell and the pressure cell filled with a supercritical water sample at 653.15 K and 25 MPa with an x-ray photon energy of 9.5 keV with a beam size of 1 *μ*m at the XPP instrument at LCLS.

The sp-XPCS has strict requirements on the optimal sample-detector geometry (see Sec. II), which limits the achievable signal-to-background ratio. In our commissioning measurements with the pressure cell, performed at the XPP instrument at LCLS, we compared the background scattering of the empty pressure cell to the scattering of the cell with a 954 *μ*m-thick supercritical H_2_O sample at 653.15 K and 25 MPa. The x-ray photon energy was 9.5 keV with an x-ray beam size of 1 *μ*m. The sample-to-detector distance was 2 m, and the detector pixel size was 50 *μ*m. The comparison of the scattering intensity for the empty cell and the cell with the SCF sample is shown in Fig. 9. The scattering of the empty cell is less than 4 × 10^−4^ photon/pixel/pulse over a wide *Q*-range. The signal-to-background ratio ranges between 4 and 5 over the whole area detector ($Q\in \left[0.02,0.15\right]$ Å^−1^). These results demonstrate that the pressure cell is able to provide a high signal-to-background ratio at low scattering angles for experiments on supercritical water.

**FIG. 10.**
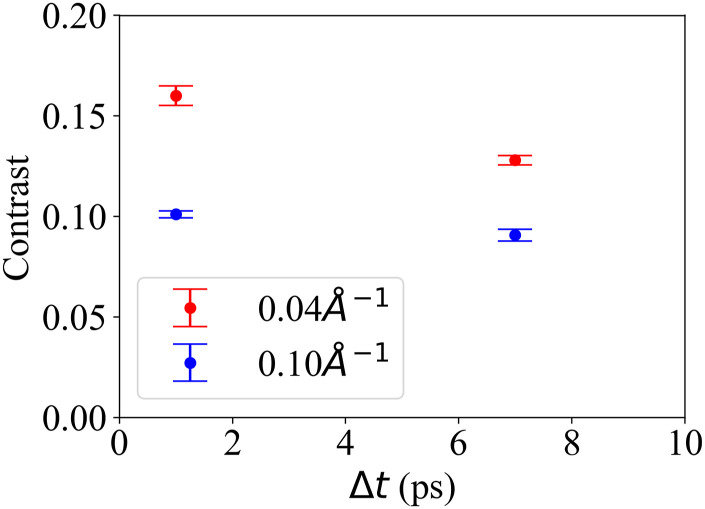
X-ray speckle contrast of x-ray pulse pairs for Δ*t* of 1 and 7 ps at *Q* of $0.04\;\;{\overset{{}^{\circ}}{A}}^{-1}$ and $0.1\;\;{\overset{{}^{\circ}}{A}}^{-1}$ for the 954 *μ*m-thick supercritical H_2_O sample at 653.15 K and 25 MPa with an x-ray photon energy of 9.5 keV and a beam size of 1 *μ*m at the XPP instrument at LCLS.

Following the signal-to-background measurements, we performed sp-XPCS measurements of supercritical H_2_O at the same experiment condition for two delay times, Δ*t* = 1 and 7 ps. At each delay time, the elastic x-ray diffraction intensities for x-ray pulse pairs were collected over 30 min with a pulse repetition rate of 120 Hz and an average pulse energy of 0.3 *µ*J per pulse pair. For simplicity of analysis, we divided the area detector into two *Q*-regions, respectively, covering $Q\in \left\{0.02,0.06\;\;{\overset{{}^{\circ}}{A}}^{-1}\right\}$ and $Q\in \left\{0.06,0.12\;\;{\overset{{}^{\circ}}{A}}^{-1}\right\}$. We refer to these regions as $Q=0.04\;\;{\overset{{}^{\circ}}{A}}^{-1}$ and $Q=0.1\;\;{\overset{{}^{\circ}}{A}}^{-1}$, respectively. The measured x-ray speckle contrasts are shown in Fig. 10. By measuring the x-ray speckle contrast for additional *Q* and Δ*t*, one can then derive the ISF using Eq. (1).

**FIG. 11.**
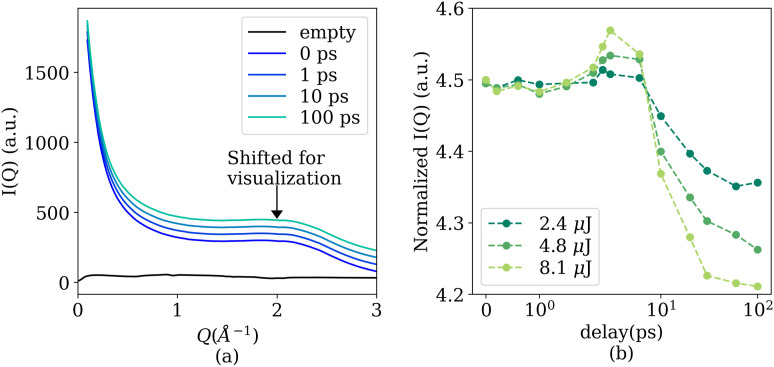
(a) Mean scattering intensity, *I*(*Q*), as a function of angular wave numbers, *Q* (Å^−1^). The black curve shows the scattering signal from an empty pressure cell, while the other curves shows the scattering signal level with H_2_O in the pressure cell at 645 K and 23 MPa with a thickness of 800 *μ*m. The legend 0, 1, 10, and 100 ps refers to the delay time between the two x-ray pulses in this XPXP measurement. The curves for 0, 1, 10, and 100 ps are shifted vertically by 0, 50, 100, and 150, respectively, with respect to their original value for a better visualization. The average pump and probe pulse energy are 5 and 8.15 *μ*J, respectively, with an x-ray photon energy of 10 keV. (b) Total scattering intensity within $Q\in \left[0.14,0.4\;\;{\overset{{}^{\circ}}{A}}^{-1}\right]$ normalized by the total scattering intensity within $Q\in \left[2.0,2.7\;\;{\overset{{}^{\circ}}{A}}^{-1}\right]$ as a function of delay time between the pump pulse and probe pulse. The probe pulse has, on average, 8.15 *μ*J, while the pump pulse energy is shown in the legend.

The signal-to-background ratio can be much higher in experiments such as XPXP measurements, where the sample thickness and detector geometry are less constrained. Figure 11(a) shows the background scattering signal with an empty pressure cell and the scattering signal with the H_2_O sample in the pressure cell in a XPXP measurement at SACLA/Spring8. H_2_O was maintained at 645 K and 23 MPa with a nominal thickness of 800 *μ*m. The sample detector distance is 14.12 cm, and the pixel size is 50 *μ*m. A tungsten beamstop with a diameter of 1 mm is installed right behind the pressure cell. This greatly reduces the static background signal from air scattering. The photon energy is 10 keV, and the average pump and probe pulse energy in Fig. 11(a) are, respectively, 5 and 8.15 *μ*J and different colors denote the different delay times between the two x-ray pulses. Because scattering intensities for different delay times show differences that are only visible though self-reference normalization, which are shown in Fig. 11(b), the scattering signals from H_2_O in Fig. 11(a) are shifted vertically for better visualization. The curves for 0, 1, 10, and 100 ps, are shifted by a numerical value of 0, 50, 100, and 150, respectively, in Fig. 11(a).

The inbuilt scintillator greatly facilitates the alignment of the pump and probe x-ray pulses. This allows us to explore multiple temperature and pressure conditions during this 60 h beamtime. For each temperature–pressure condition, we performed XPXP measurements at multiple delay times. For 645 K and 23 MPa, shown in Fig. 11(b), a total of 13 delay times were measured between 0 and 100 ps. We normalized the scattering intensity *I*(*Q*) based on the average scattering intensity of $Q\in \left[2.0,2.7\;\;{\overset{{}^{\circ}}{A}}^{-1}\right]$. The averaged normalized intensity of $Q\in \left[0.14,0.4\;\;{\overset{{}^{\circ}}{A}}^{-1}\right]$ is shown in Fig. 11(b) as a function of different delay times between the pump and probe x-ray pulses for three pump pulse energies, 2.4, 4.8, and 8.1 *μ*J. The probe pulse has an average pulse energy of 8.15 *μ*J. This self-normalized measurement shows unequivocally an increase in the scattering signal within 10 ps and has a clear dependency on the pump pulse energy. This time and length scale is close to that of the lifetime and spatial extend of generic molecular clusters near the critical point.^14^ Therefore, we believe that the observed structure factor change reflects the destruction and reorganization of the molecular clusters induced by the strong x-ray pump pulses. Detailed analysis is ongoing to determine the origin of the observed phenomena.

## V. CONCLUSIONS

The advent of x-ray FELs has enabled the development of ultrafast multi-pulse x-ray scattering techniques, such as sp-XPCS and XPXP measurements, which have opened exciting new opportunities to characterize nano-scale ultrafast dynamic processes occurring in SCFs. In the present work, we introduce a pressure cell design that is optimized for conducting sp-XPCS and XPXP experiments on SCFs. The pressure cell is able to create and contain samples of numerous SCFs, including substances with high critical temperature and pressure, such as water or dodecane. The cell is designed to maintain thermodynamic conditions precisely over the long duration of x-ray FEL experiments. The pressure cell has the following features:

1. construction from titanium for chemical inertness and machining using single point diamond turning to reduce requirements for manual processing and polishing;
2. provide visible and x-ray optical access to the SCF sample by 100 *μ*m-thick single crystal diamond windows;
3. maximum design pressure of 30 MPa and maximum design temperature of 675 K, with a factor of safety of 1.9;
4. low scattering background, typically yielding a signal-to-background ratio of 4–5 at low scattering angle with supercritical water;
5. adjustable geometry of the sample and detector to optimize SNR during sp-XPCS measurements; and
6. incorporation of *in situ* diagnostic into the pressure cell to monitor beam overlap.

We provide a detailed description of the pressure cell and a procedure to achieve reliable operation when using SCF samples. The CAD files for this apparatus are included in the present article as the supplementary material. The apparatus has been commissioned and used for sp-XPCS, XPXP, WAXS, and SAXS experiments on SCFs. It has been demonstrated to have exceptional temperature and pressure stability during extended acquisition periods required for experimental multi-pulse, ultrafast measurements of SCFs at x-ray FELs.

## SUPPLEMENTARY MATERIAL

The complete CAD file of the pressure cell, including the pressure cell body, cone, and the retaining nut, is provided in the STEP format to facilitate the verification and utilization for other researchers.

## Figures and Tables

**FIG. 12.**
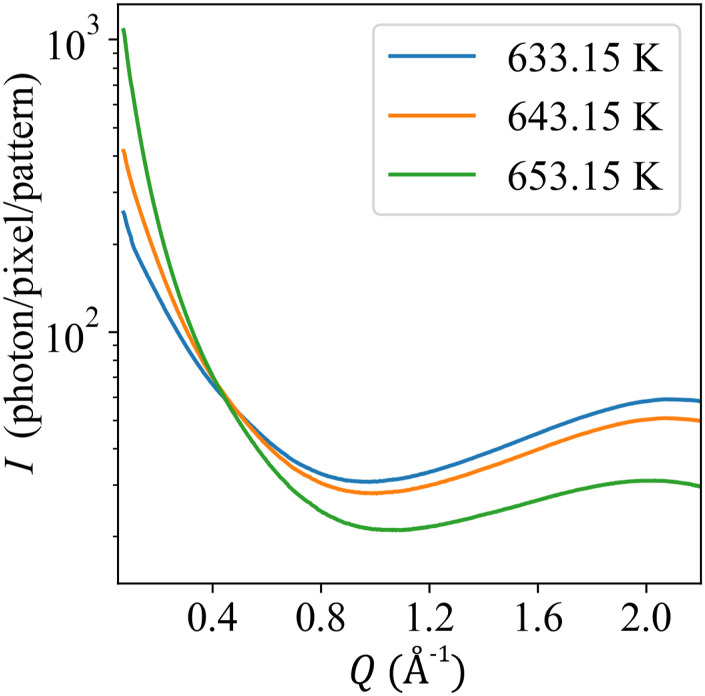
Measurements of the averaged x-ray scattering intensity of supercritical water at different temperatures and a pressure of 25 MPa. These data were acquired at the BL4-2 beamline at SSRL with a photon energy of 15 keV and a photon flux of 2.5 × 10^12^ photons per second.

**FIG. 13.**
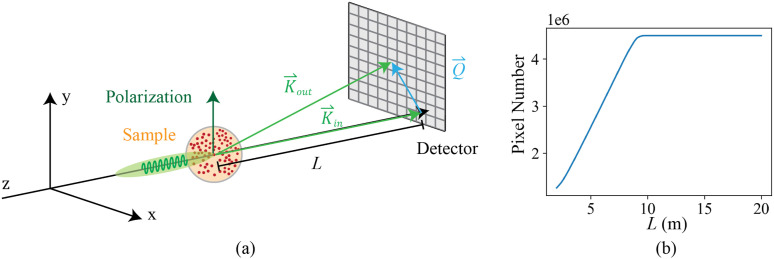
(a) Model to calculate the angular wave-vector $\vec{Q}$ for each pixel on the area detector. (b) Pixel number for different detector distance *L* at an incident x-ray photon energy of 9.5 keV. We assume a 1500 × 1500 pixel detector, with a pixel size of 50 *μ*m. The center of the area detector is assumed to be positioned $Q=0.1\;\;{\overset{{}^{\circ}}{A}}^{-1}$. We count the pixel number within $Q\in \left[0.08\;\;{\overset{{}^{\circ}}{A}}^{-1},\;0.12\;\;{\overset{{}^{\circ}}{A}}^{-1}\right]$ for each *L*.

**FIG. 14.**
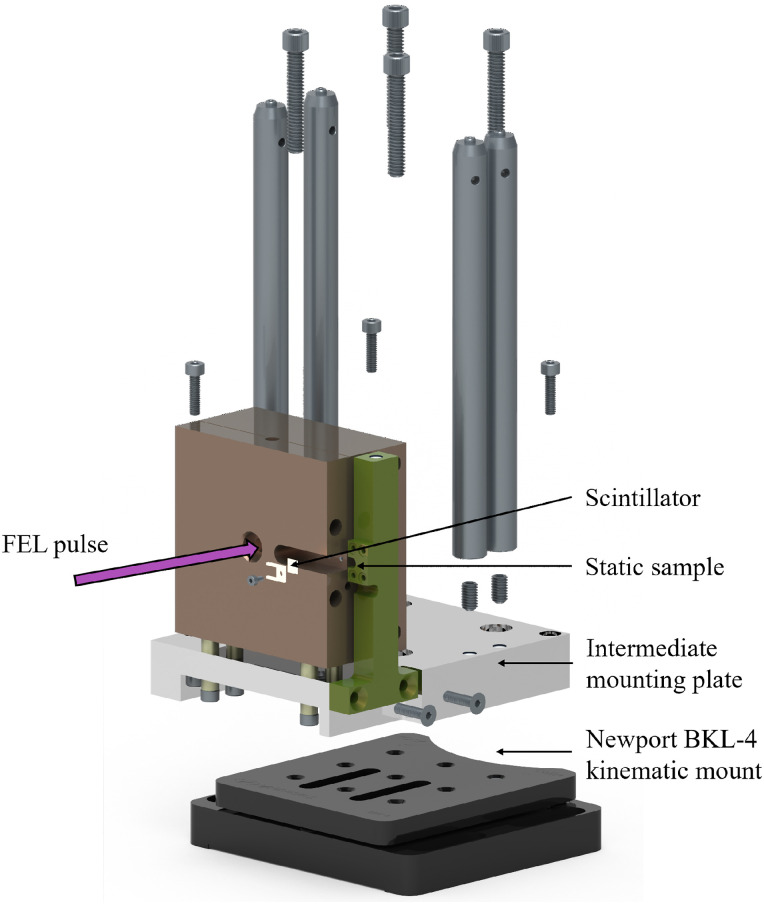
Pressure cell assembly as typically used during measurements. X-ray FEL pulses are traveling from left to right. The pressure cell is shown in golden-brown, the scintillator and its holder are shown in in white, and the static sample and its holder are shown in in green. Vertical optical posts are used to keep the electrical cable and piping out of the beam path. An aluminum intermediary plate (gray) is used to attach all these components to a Newport kinematic mount (black), which interfaces with the beamline. Four ceramic posts are located between the cell and the aluminum plate to thermally insulate the cell and improve its thermal stability. Ceramic fiber paper is also wrapped around the cell for thermal insulation (not shown here).

**FIG. 15.**
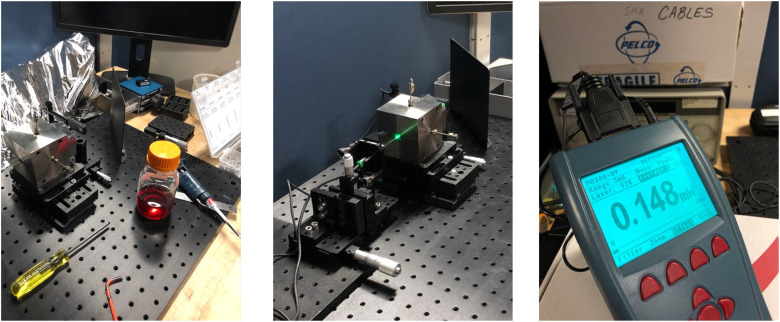
Setup for sample thickness measurements. (left) Dye used for absorption measurements. (middle) Laser absorption through the entrance optical hole in the pressure cell. (right) The intensity of photo-diode placed at the exit optical hole in the pressure cell.

## Data Availability

Data sharing is not applicable to this article as no new data were created or analyzed in this study.

## APPENDIX A: PRESSURE SAFETY ANALYSIS

The metal cell body and cone is designed with sufficient materials thickness (at least 5 mm) to prevent mechanical failure during an over-pressurization event. The main point of failure of the pressure cell, besides the diamond windows, is the threads on the retaining nut. We followed the analysis of Brooks *et al.*^38^ to ensure that the shear stress experienced by these threads is less than the shear strength of the titanium alloy,

$$\frac{F}{A_{s}}<\sigma_{max,shear},$$ (A1)

$$A_{s}=\pi nL_{e}K_{n,\max}\left[\frac{1}{2n}+0.577\;35\left(E_{s,\min}-K_{n,\max}\right)\right],$$ (A2)

where *F* is the maximum axial load, *A*_*s*_ is the shear area, *n* = 667 threads/m is the number of threads per unit length, *L*_*e*_ > 5 mm is the engagement length of the thread, *K*_*n*,max_ = 48.7 mm is the maximum minor diameter of the internal thread, and *E*_*s*,min_ = 48.9 mm is the minimum pitch diameter of the external thread. To calculate the maximum axial load, we used $F=\frac{T}{c\;D}$, where *c* is the coefficient of friction for Ti 6-Al-4V, *D* is the major diameter of the threads, and *T* is the torque required to tighten the retaining nut into the main body of the pressure cell. For *c* = 0.3, *D* = 50 mm, and *T* = 300 Nm, the axial load, *F*, is 20 kN. For a ductile material, such as grade 5 Ti-6Al-4V alloy, the shear strength is estimated to be 0.58 times its tensile strength, *σ*_max,shear_ ≈ 880 MPa. This corresponds to a safety factor of 11.3.

## APPENDIX B: DIFFRACTION CROSS SECTION

To estimate the x-ray scattering intensity for various sample thicknesses and detector distances, we performed SAXS measurements of supercritical water at 25 MPa at different temperatures at SSRL (beamline BL4-2) with a photon energy of 15 keV and a photon flux of 2.5 × 10^12^ photons per second. The sample thickness was *d* = 400 *µ*m with a detector-sample distance of 31.6 cm and a detector pixel size of 172 *µ*m.

The background-subtracted and averaged scattering intensities are shown in Fig. 12 for different sample temperatures. We have performed the analysis presented in Sec. II at $Q=0.1\;\;{\overset{{}^{\circ}}{A}}^{-1}$ and 653.15 K. The Thomson scattering cross section (*dσ*/*d*Ω)_Th_ is computed using Eq. (3). In Eq. (3), *d*Ω is taken to be the solid angle spanned by each pixel.

## APPENDIX C: DETECTOR PIXEL NUMBER

We estimate the pixel number by counting the number of pixels with angular wave-vectors within the target *Q*-range on an area detector. For simplicity, we assume that the area detector is orthogonal to the incident x-ray propagation direction ${\vec{K}}_{\text{in}}$ [Fig. 13(a)]. The overall beamline geometry is depicted in Fig. 13(a). For a given x-ray detector distance *L* and detector pixel size, one can calculate the angular wave-vector $\vec{Q}={\vec{K}}_{\text{out}}-{\vec{K}}_{\text{in}}$ for each pixel. To maximize the pixel number within the target *Q*-range, the central pixel of the detector is placed at an angular wave-vector $\vec{Q}$ at the center of the target *Q* range.

For the pixel number estimation used in Sec. II, we assume that the detector has a pixel size of 50 *µ*m and a resolution of 1500 × 1500 pixel. For each sample–detector distance *L*, we first calculate the angular wave-vector $\vec{Q}$ for each pixel and then count the pixel number within $Q\in \left\{0.08\;\;{\overset{{}^{\circ}}{A}}^{-1},\;0.12\;\;{\overset{{}^{\circ}}{A}}^{-1}\right\}$. The corresponding pixel number for each *L* is reported in Fig. 13(b) and was used in Eq. (2) to obtain the SNR reported in Fig. 2(c).

## APPENDIX D: BEAMLINE INSTALLATION

Beamtime at FEL facilities is currently a very limited resource. This pressure cell therefore requires a high level of reliability and must accommodate rapid field repairs if any unexpected issues were to occur during an experiment. The high working temperature of the cell is a major obstacle for these field repairs, as the first step in repairs is disassembly from the beamline. To address the high temperature, four 1/4 in. −20 threading holes are included on the intermediate mounting plate in Fig. 14. This allows users to install two long 1/2 in. standard optical posts as a safe handle to remove the pressure cell from the beamline even with the pressure cell at high temperature, as shown in Fig. 14. To facilitate the installation and to increase the portability, the pressure cell assembly can be installed on a Newport BKL-4 kinematic mount (shown in Fig. 14), commonly found at a number of lightsources, or to a motion stage through three M4 screws. The fluid lines and cartridge heaters are also equipped with quick-disconnect connectors. After removal, the cell can be quickly cooled down in water so that leaks or failures can be addressed.

The static sample assembly (green in Fig. 14) holds a static sample of silica nanopowder coplanar with the YAG scintillator screen and the mid-plane of the sample. This static sample is used to determine the effective overlap *μ* within a pulse pair [Eq. (1)].^21^ Similar to the YAG scintillator screen, it can be quickly accessed by a transverse translation in order to perform regular calibration over the course of an experiment.

## APPENDIX E: MOTION AXES

The depth of field of the imaging system presented in Fig. 6 is only 6.2 *µ*m. A precise, repeatable, and motorized motion system is therefore required to align the SCF sample, the static sample, and the YAG scintillator screen to the FEL pulses and the imaging system and to allow switching between these different devices. Within the coordinate system defined in Fig. 6(a), two linear motorized stages are required for the *x*- and *y*-motion of the pressure cell to adjust the relative position between the pressure cell and the x-ray pulse, and a 50 mm travel range is required for the *x*-axis in order to cover the sample, the scintillator screen, and the static sample. In addition, three linear stages are required for the *x*, *y*, *z* motion of the x-ray beam profile monitor with respect to the pressure cell to optimize the imaging quality of the x-ray beam profile monitor on the scintillator screen. Due to the limited working distance of the imaging system (Fig. 6), the right angle mirror also requires two manual *x* and *z* stages to adjust its position with respect to the x-ray beam profile monitor. In this way, one can optimize the distribution of the working distance between *l*_1_ and *l*_2_ (Fig. 6) for an optimal heat insulation between the lens and the cell, necessary to avoid thermal distortion artifacts in the imaging system.

## APPENDIX F: LASER ATTENUATION MEASUREMENT OF SAMPLE THICKNESS

X-ray attenuation measurements are not always possible to characterize the sample thickness. One can also use visible lasers to measure the sample thickness using a laser attenuation measurement. This was done by measuring the transmission of laser light through the fluid sample using a photo-diode or a commercial laser power-meter. Our sample thickness measurement setup is shown in Fig. 15.

A 520 nm laser was used to measure the laser intensity attenuation. To obtain reliable attenuation measurements, we tightly focus the laser beam on the pressure cell to a spot size smaller than the optical entrance, which is 1 mm in diameter. Since water does not significantly absorb light at 520 nm, a [Fe(phen)_3_]SO_4_ dye (Ferroin) was dissolved in water. The extinction coefficient (*ɛ*) of this aqueous solution was measured as 11 210 M^−1^ cm^−1^ by ultraviolet visible spectroscopy. The sample thickness can be determined following

$$I=I_{0}\exp\left\{-\varepsilon cd\right\},$$ (F1)

where *I*_0_ and *I* are the laser intensity before and after the sample, respectively, and *c* is the dye concentration. We used the photo-diode intensity measured with pure water as *I*_0_, thereby accounting for the transmissivity of the diamond windows. To determine the sample thickness, we measured the transmissivity with multiple dye concentrations. A sample thickness of 440 ± 35 *μ*m is obtained for a cone designed with a nominal sample thickness of 600 *μ*m. The measured deviation from the nominal value is induced by a combination of the machining error and the deformation of the metal parts under the large preloaded force on the retaining nut.

## APPENDIX G: MECHANICAL PROPERTIES

The mechanical properties of materials utilized in the construction are shown in Table III.

**TABLE III.** Mechanical properties of materials utilized in the construction of the pressure cell.

Property	Ti-6Al-4V^a^	Type IIa CVD diamond^b^
Density (g/mm^3^)	4.43	3.515
Hardness, Knoop	363	90 GPa
Tensile strength, yield (MPa)	880	500–1400
Modulus of elasticity (GPa)	113.8	1050
Compressive yield strength (MPa)	970	>110 000
Fracture toughness (MPa m^1/2^)	75	
Poisson’s ratio	0.342	0.1–0.29
Shear modulus (GPa)	44	
Shear strength (GPa)	550	

^a^ Properties are from the ASM Aerospace Specification Metals, Inc. website.

^b^ Properties are from the single-crystal CVD diamond data sheet of Applied Diamond USA.
